# Risk factors for recanalization of truncal veins following endoluminal ablation

**DOI:** 10.1016/j.jvsv.2024.101860

**Published:** 2024-02-28

**Authors:** Matthew Vuoncino, Joel Harding, Nasim Hedayati, Mimmie Kwong

**Affiliations:** Division of Vascular Surgery, The University of California Davis, Sacramento, CA

**Keywords:** Recanalization, Registry, Venous

## Abstract

**Background:**

Recanalization of target veins after treatment of superficial venous incompetence has clinical implications and may depend on the type of intervention. The aim of this study was to evaluate patient and procedural factors associated with truncal vein recanalization in a large study cohort using the Vascular Quality Initiative (VQI) Varicose Vein Registry.

**Methods:**

We performed a retrospective review using the VQI Varicose Vein Registry from 2014 to 2018. We evaluated all procedures performed for truncal venous insufficiency. Demographic data and information about treatment modality were collected. Patients were separated into recanalization and nonrecanalization groups based on the status of the treated vein at follow-up ultrasound examination. The vein was only considered recanalized if the VQI noted complete recanalization of the target vein. Univariate and multivariate comparisons were performed as appropriate.

**Results:**

A total of 10,604 procedures were performed in 7403 patients. The average age was 55.9 years and 70.3% of the patients were female. Patients with recanalization were more likely to have a history of phlebitis (*P* < .001) and had a higher mean body mass index (30.5 vs 32., kg/m^2^ ; *P* = .006) compared with those without recanalization. There was no difference in the use of compression therapy, anticoagulation, deep venous reflux, number of pregnancies, prior deep vein thrombosis, Venous Clinical Severity Score, and clinical-etiology-anatomy-pathophysiology between patients with and without recanalization. The number of truncal veins treated per procedure was higher in the recanalization group compared with the nonrecanalization group (2.36 vs 1.88; *P* = .001). After multivariate logistic regression, laser ablation was associated with higher rate of recanalization compared with radiofrequency ablation (*P* = .017).

**Conclusions:**

This study is the first to use VQI based data to describe risk factors for recanalization following treatment of truncal venous reflux. The use of laser ablation for truncal veins is associated with a higher risk for recanalization compared with radiofrequency ablation. Obesity, prior phlebitis, and number of veins treated were independently associated with increased rate of recanalization.

**Article Highlights**

• **Type of Research:** Registry-based retrospective cohort study
• **Key Findings:** The use of laser ablation for truncal veins is associated with a higher risk for recanalization compared with radiofrequency ablation. Obesity, prior phlebitis, and number of veins treated were independently associated with an increased rate of recanalization.
• **Take Home Message:** The Vascular Quality Initiative Varicose Vein Registry data suggests laser ablation is associated with higher rates complete recanalization compared with radiofrequency ablation.

Treatment for superficial venous insufficiency has undergone a paradigm shift over the last two decades. Surgical stripping or ligation, which often required general anesthesia or sedation, has been replaced with office-based, percutaneous interventions.^1^ Noninvasive procedures such as endovenous laser therapy (EVLT), radiofrequency ablation (RFA), and ultrasound-guided foam sclerotherapy, among other noninvasive treatment options, have become the primary modalities for the endoluminal treatment of refluxing veins.

For these percutaneous procedures, the primary goal is to achieve durable occlusion of the target vein. Energy transmitted during RFA and EVLT induces endothelial damage, vessel contraction, and destruction of the incompetent vein. Venous outflow from an extremity is then directed to a path with competent valves. Recanalization of a treated vein can cause symptom recurrence and has been associated with delayed would healing in venous leg ulcers.^2^^,^^3^ Prospective European studies have demonstrated increased risk of recanalization with ultrasound-guided foam sclerotherapy, but no difference between EVLT and RFA.^4^^,^^5^ In the United States, previous single center studies have shown that increased body mass index (BMI) and target vein diameter are associated with recanalization of the target vein.^6^^,^^7^ However, there is paucity of data regarding the risk factors associated with recanalization of superficial veins for U.S. patients on a national practice scale. This is important to evaluate as studies have demonstrated wide variations in practice patterns for vein treatments throughout the different regions in the United States, thereby limiting the generalizability of single-center studies.^8^ The aim of this study was to determine patient and procedural factors associated with truncal vein recanalization in a large multi-institutional, nationwide cohort using the Vascular Quality Initiative (VQI) Varicose Vein Registry (VVR).

## Methods

The VQI VVR was founded in 2014 with aims to capture venous procedures performed nationwide in both the outpatient and inpatient setting. Data were collected from 18 regional groups and >900 participating centers. Protocol approval was obtained by both the local institutional and national VQI review boards before data collection. The institutional review board committee reviewed the study and determined that it was exempt given the deidentified nature of the VQI data. We performed a retrospective review of the prospectively collected VQI VVR data from 2014 to 2018 to identify all cases of truncal venous insufficiency that were treated. Demographic data and information about treatment modality were collected. Given the time frame during which the data were collected, data regarding cyanoacrylate, proprietary endovenous microfoam, and mechanicochemical ablation were not available to be included in this analysis. Details concerning the patient, procedural, and long-term follow-up data collected by the VVR have been described previously.^9^

Analysis was limited to patients with interventions performed on truncal veins. Truncal veins, as defined by the VQI VVR, are major axial superficial veins in the thigh or leg, including the great saphenous vein (GSV), superficial accessory GSV, anterior accessory GSV, and small saphenous vein. Patients were separated into recanalization and nonrecanalization groups based on the status of the treated vein at follow-up ultrasound examination. Recanalization categories in the VQI VVR include none, partial, complete, or unknown. In this study, a truncal vein was only considered recanalized if the VQI listed complete recanalization of the target vein. Veins that had partial or no recanalization were not considered recanalized. Veins that were not imaged or for whom recanalization outcome was not recorded were recategorized as missing. Compression therapy use before the procedure was quantified by the VQI VVR dataset as none, intermittent, most days, and always. In this study, patients who reported compression use as intermittent and most days were combined into a single group designated as sometimes. If a patient had multiple limbs treated that were captured within the registry, each limb was captured as a different case. Univariate and multivariate statistical analyses were performed using Stata (StataCorp SE, College Station, TX) to compare patient characteristics, procedural details, and postprocedural outcomes between the groups. A *P* value of <.05 was considered significant for all analyses.

## Results

During the study period, from 2014 to 2018, 17,908 procedures were captured by the VVR. There were 10,604 procedures on truncal veins performed in 7403 patients. The average age was 55.9 years and 70.3% of the patients were female (Table I). Of the patients, 79.9% were White, 6.2% were Black, 1.2% were Asian, and 12.1% were of unknown race. The average BMI for the cohort was 30.4 kg/m^2^. A history of phlebitis and lower extremity deep vein thrombosis was present in 9.3% and 4.5% of patients, respectively. Prior varicose vein treatment (VVT) had been performed in in 21.1% of patients. For right-sided treatment, leg compression was not used in 31.3% of cases, sometimes used 34.2% of cases, and always used 34.4% of cases. For left-sided treatment, leg compression was not used in 32.5% of cases, sometimes used 33.7% of cases, and always used 33.8% of cases. The types of anesthesia used were general (18.2%), conscious sedation (41.6%), and local/regional (83.5%). Compression use averaged 8.5 days in duration before intervention. The most common intervention performed was RFA (59.7%), followed by EVLT (32.5%), phlebectomy (4.14%), stripping (2.04%), and foam ablation (0.21%) (Fig). Anticoagulation was being taken by 18.0% of patients at the time of intervention.

**Table I:** Patient factors

Patient factors	Total	%
Age, years (mean)	55.9	
Gender		
Male	3155	29.75
Female	7449	70.25
Procedural year		
2014	65	0.61
2015	2472	23.31
2016	3827	36.09
2017	3490	32.91
2018	750	7.07
Race		
Native American	22	0.21
Asian	129	1.22
Black	659	6.21
White	8480	79.97
Unknown	1281	12.08
Mean BMI (kg/m)	30.4	
History of phlebitis	986	9.30
History of prior DVT	478	4.51
Prior varicose vein treatment	2235	21.08
Mean No. of pregnancies	2.22	
Mean VCSS		
Right	7.87	
Left	7.98	
Mean CEAP		
Right		
0	2081	18.1
1	380	3.31
2	3300	28.7
3	3185	27.7
4a	1630	14.2
4b	322	2.80
5	210	1.83
6	373	3.25
Left		
0	2042	17.8
1	361	3.14
2	3247	28.3
3	3253	28.3
4a	1620	14.1
4b	320	2.79
5	243	2.11
6	373	3.52
Compression use		
None	2894	27.29
Sometimes	3076	29.01
Always	3092	29.16
Preprocedural anticoagulation		
None	9623	90.75
Yes, held	822	7.75
Yes, continued	159	1.50

*BMI*, Body mass index; *CEAP*, clinical-etiology-anatomy-pathophysiology; *DVT*, deep vein thrombosis; *VCSS*, Venous Clinical Severity Score.

**Fig:**
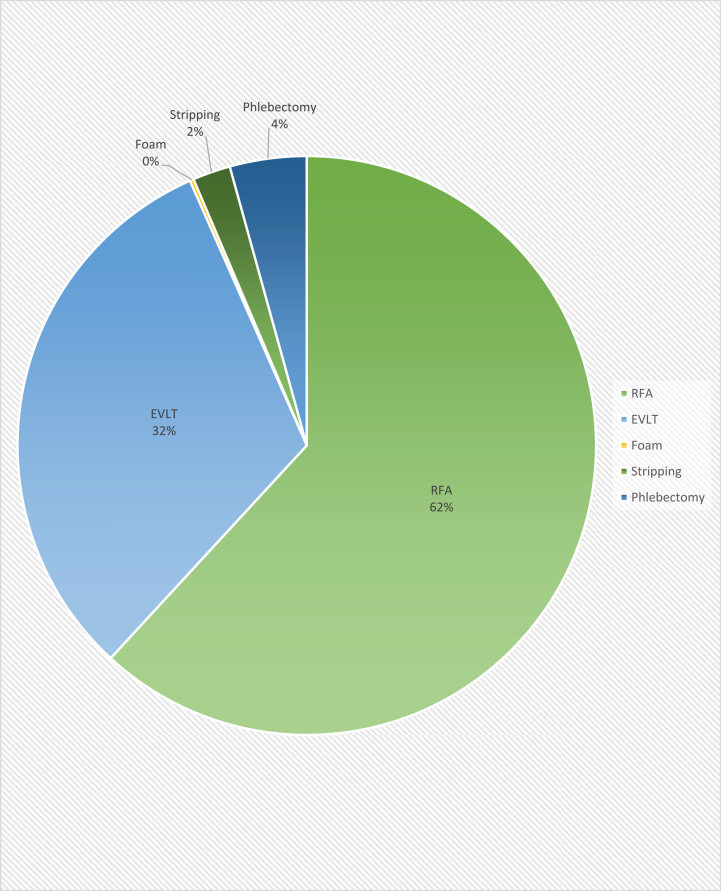
Distribution of procedure types. *EVLT*, Endovenous laser therapy; *RFA*, radiofrequency ablation.

The mean follow-up was 142 ± 170 days. Of note, the average follow-up time for patients with recanalization was 184 days compared with 112 days in patients without recanalization (*P* = .002). Of the patients, 8.29% were using anticoagulation at the time of follow-up. At follow-up, compression use was always used, sometimes used, and not used in 34.4%, 34.2%, and 31.3%, respectively, of patients who underwent right-sided treatments and 33.8%, 33.6%, and 32.5%, respectively, of patients who underwent left-sided treatments. At the time of the procedure, the mean clinical-etiology-anatomy-pathophysiology (CEAP) score on the left-sided treatment legs was 3.26 and 3.22 on right-sided treatment legs. The mean Venous Clinical Severity Score (VCSS) was 8.14 on left-sided treatment legs vs 8.03 on right-sided treatment legs. At follow-up the average CEAP was 2.21 and 2.24 on the left and right, respectively, and the average VCSS was 3.70 and 3.75 on the left and right, respectively.

Of the 7403 patients with truncal interventions, 83 (1.1%) had the treated veins recanalize. Patients with recanalization were more likely to have a history of phlebitis (*P* < .001) and had a higher mean BMI (32.7 vs 30.5 kg/m^2^; *P* = .006) compared with those without recanalization. There was no difference in age (56.4 vs 54.6; *P* = .25), gender (*P* = 0. 88), race (*P* = .25), the use of compression therapy after intervention (*P* = .49), deep venous reflux (*P* = .59), number of pregnancies (2.2 vs 2.2; *P* = .63), prior deep vein thrombosis (*P* = .97), prior VVT (*P* = .57), general anesthesia (*P* = .95), VCSS L (8.1 vs 8.1; *P* = .91), and VCSS R (8.0 vs 8.8; *P* = .21) between patients with and without recanalization (Table II). CEAP values, when compared as categorical variables, were not associated with recanalization on the right (*P* = .39) or the left (*P* = .84). Preprocedural anticoagulation (*P* = .32), periprocedural anticoagulation (*P* = .62), and postprocedural anticoagulation (*P* = .91) were not significantly associated with recanalization The diameter of the target vein was not significantly larger in the recanalized patients compared with the nonrecanalized (6.98 mm vs 7.58 mm; *P* = .29), nor was the target vein length (*P* = .71). The number of truncal veins treated per procedure was higher in the recanalization group compared with the nonrecanalization group (2.36 v 1.88; *P* = .001). On univariate analysis, laser ablation (*P* = .002) and RFA (*P* = .001) were associated with recanalization (Table III). Differing levels of maximum energy (*P* = .13) and maximum power (*P* = .37) were not associated with recanalization in patients treated with laser ablation. Similarly, differing levels of minimum energy (*P*= .16) and minimum power (*P* = .37) were not associated with recanalization in laser ablation cases. Laser wavelength was assessed as categorical variables. The VVR had inputs for wavelength ranges as follows: 800 to 1000 nm, 1300 to 1400 nm, and 1400 to 1500 nm. There was no association between wavelength and recanalization of truncal veins (*P* = .43). Following step-wise, multivariate logistic regression, BMI (odds ratio [OR], 1.04; 95% confidence interval [CI], 1.01-1.07), number of veins treated (OR, 1.37; 95% CI, 1.17-1.61), EVLT (OR, 2.27; 95% CI, 1.45-3.56), and prior phlebitis (OR, 3.44; 95% CI,2.03-5.83) were associated with recanalization (Table IV).

**Table II:** Comparison of patient factors in recanalized vs nonrecanalized patients

Patient factors	Nonrecanalized	Recanalize	*P* value
Age, years	56.4	54.6	.25
Gender			.88
Male	29.6%	28.9%	
Female	70.3%	71.8%	
Race			.25
Native American	0.2%	19.0%	
Asian	1.2%	1.2%	
Black	6.7%	6.8%	
White	82.8%	82.8%	
Unknown	8.8%	8.7%	
BMI	30.500	32.700	.01
History of phlebitis	8.5%	23.5%	<.001
History of prior DVT	4.9%	4.8%	.97
Prior varicose vein treatment	26.6%	23.5%	.57
Mean No. of pregnancies	2.2%	2.3%	.63
Mean VCSS			
Left	8.14	8.14	.54
Right	8.02	8.02	.21
CEAP			
Left	3.25	3.17	.67
Right	3.08	3.23	.52
No. of veins treated	1.88	2.36	<.001
Preprocedural compression			.25
None	99.0%	1.0%	
Sometimes	98.2%	1.4%	
Always	99.3%	0.8%	
Preprocedural anticoagulation			.32
None	98.9%	1.1%	
Held	98.4%	1.7%	
Continued	100.0%	0.0%	

*BMI*, Body mass index; *CEAP*, clinical-etiology-anatomy-pathophysiology; *DVT*, deep vein thrombosis; *VCSS*, Venous Clinical Severity Score.

**Table III:** Comparison of recanalization rates by procedure type

Procedure type	No.	Recanalized, No. (%)	Nonrecanalized, No. (%)	*P* value
RFA	3999	31 (0.78)	3968 (99.22)	.002
Laser	2908	48 (1.7)	2860 (98.3)	.001
Foam	21	1 (4.8)	20 (95.2)	.211
Stripping	96	0 (0)	96 (100)	.629
Phlebectomy	289	8 (2.8)	281 (97.2)	.007

*RFA*, Radiofrequency ablation.

**Table IV:** Multivariate regression of factors associated with recanalization

	OR	CI
BMI	1.04	1.01-1.07
No. veins treated	1.37	1.17-1.61
EVLT	2.27	1.45-3.56
Phlebitis	3.44	2.03-5.83

*BMI*, Body mass index; *CI*, confidence interval; *EVLT*, endovenous laser therapy; *OR*, odds ratio.

## Discussion

Venous insufficiency can contribute to significant patient morbidity including pain, heaviness, aching, pruritis, and nonhealing wounds that may greatly disrupt a patient's life. The goal of intervention for superficial venous reflux is to eliminate blood flow through an incompetent target vein contributing to the patient's symptoms. Recurrence can result in resurgence of symptoms, delayed wound healing, or redevelopment of a venous wound. Therefore, durable occlusion with the absence of recanalization is a primary goal of intervention. Our study harnesses one of the largest, nationwide databases of venous procedures and identified a number of factors associated with recanalization.

In our study, a higher BMI conferred a higher risk of recanalization. For each 1 kg/m^2^ increase in BMI, there was a 4% increase the odds of developing recanalization. Prior studies have noted a similar relationship.^10^ Indeed, BMI has been linked to the development of venous insufficiency.^11^ Theories for this correlation include a connection between body weight and obstructive venous pathology, with increased central pressure resulting in decreased venous return and increases distal/extremity pressure.^12^ A multicenter retrospective study assessing 249 limbs for recanalization after RFA found a higher mean BMI in the recanalized group (33.5 kg/m^2^vs 30.3 kg/m^2^), although this difference was not statistically significant.^13^ Interestingly, a single-center retrospective study performed by Ahmed et al^7^ found BMI to be correlated with increased recanalization after treatment of perforator veins. However, in their study, BMI was not associated with increased recanalization in truncal veins.^7^

In our study, we noted that anticoagulation use does not increase the risk of recanalization. The VQI VVR records separate inputs for anticoagulation use at different time points and includes preprocedural, periprocedural, and long-term (use at follow-up) anticoagulation. Therefore, we were able to more specifically investigate the effect of anticoagulation on recanalization. In our study, we found that anticoagulation use at any time point—before the procedure, during the procedure, OR at the time of follow-up—was not associated with recanalization. Furthermore, the type of anticoagulation patients were on before surgery and whether or not the anticoagulation was held or continued did not affect recanalization outcomes. Gabriel et al^14^ reported no change in recanalization rates in 45 patients taking warfarin at the time of endovenous ablation. However, no prior studies have evaluated the relationship between recanalization and the use of differing types of anticoagulation regimens and at varying time points.

In our cohort, patients with recanalization were more likely to have a history of phlebitis; 23.5% of the patients who experienced recanalization also had prior phlebitis as compared with only 8.5% of those without recanalization (*P* < .001). Overall the incidence of prior phlebitis was 9.3% in VVR cases from 2014 to 2018. Early reviews of the VQI VVR illustrated postintervention phlebitis among the most common complication after ablation, occurring in approximately 1% of cases.^9^ Although a known postoperative adverse event, a history of phlebitis has not been associated with recanalization previously.

The average number of truncal veins treated per case was 1.89. The number of truncal veins treated per procedure was higher in the recanalization group compared with the nonrecanalization group (2.36 vs 1.88; *P* = .001). It may be that the number of truncal veins treated is a marker of more extensive venous incompetence and this factor may belie the higher risk of recurrence. Our study did not evaluate the treatment of nontruncal veins performed during the same procedure; thus, a more thorough analysis of anatomic complexity could not be performed. Although the Society for Vascular Surgery guidelines discuss truncal interventions and concomitant interventions for associated tributaries, concomitant interventions on multiple truncal veins are not addressed in the guidelines.^15^ The current guidelines recommend the ablation of insufficient truncal veins with concomitant phlebectomy or ultrasound-guided foam sclerotherapy of associated varicosities. This pairing of interventions has shown improved quality-of-life metrics, lower VCSS pain scores, and a greater median improvement in VCSS scores.^16^, ^17^, ^18^ Treatment of venous insufficiency is often a shared decision-making process between the clinician and the patient. The decision to undergo staged vs combined procedures may account for many factors aside from anatomic complexity and may include patient risk for complications, anesthetic needs, procedure duration, anticipated postoperative pain, ad recovery time.

Our data suggest that EVLT confers an increased risk of recanalization, with patients who receive laser ablation experiencing a 2.3 times higher odds of recanalization compared with patients who do not undergo EVLT. Interestingly, published data comparing recanalization rates between EVLT to RFA demonstrate mixed findings. Yoon et al^19^ conducted a single-center experience with 246 limbs, illustrating significantly more same site recanalization with RFA compared with EVLT (2.06% vs 0.82%; *P* = .0388). Comparatively, prospective studies from Europe have shown that EVLT has similar recanalization rates to stripping and RFA.^4^^,^^5^^,^^20^ There are numerous studies that illustrate a dose-response effect of energy delivery and duration of target vein occlusion.^21^^,^^22^ Our dataset did not show an association of power and energy use with recanalization. Likely, there are additional confounding factors within the cohort that may not be captured by the VVR, contributing to our observed EVLT recanalization rates.

One major strength of our study is the use of a large, nationwide U.S. database to study risk factors for recanalization. Participating facilities can range from large to small and academic to private practices. They can include providers from differing training pathways, including vascular, cardiology, and interventional radiology. They can include patients of differing demographic and socioeconomic status who have coverage from differing insurance types. Finally, the VQI VVR captures a wide degree of technique variability between providers. As such, this study provides one of the most comprehensive views of the nationwide VVT practice available through a registry/database and perhaps one of the more accurate reflections of real-world patient outcomes in U.S. practices. However, at the same time, there are many patient and procedural factors that are not accounted for in VQI that may explain the differences seen in our study, such as patient medical comorbidities, provider-dependent variables for energy delivery, or additional procedures performed after the index operation. Finally, this study based off the VQI VVR is limited by its retrospective nature and its reliance on a registry. Data points analyzed are only the ones made available through the registry therefore it can be difficult to obtain granular data. The presence of target vein recanalization is documented at follow-up; however, the exact timing of the follow-up duplex examination is not captured by the registry specifically. Furthermore, a standardized follow-up in not implemented in our data; therefore, a wide range of follow-up durations could also be confounding recanalization rates. Being a registry, there are many variables with missing or incomplete data. Additionally, despite having access to a national database, our cohort only consists of 83 cases of recanalization; despite being the largest dataset to analyze recanalization, it may still be underpowered to assess granular questions. Furthermore, a follow-up time of 142 days is relatively short to assess for recanalization. Comparatively, the mean healing time for venous stasis wounds is 6 months.^23^^,^^24^ A prospective, multicenter study in the United States with more frequent and detailed follow-up would be able to better identify which factors specifically lead to recanalization.

## Conclusions

This study is the first to use VQI-based data to describe risk factors for recanalization after treatment of truncal venous reflux. The use of laser ablation for truncal veins is associated with a higher risk for recanalization compared with RFA. Obesity, prior phlebitis, and number of veins treated were also associated independently with an increased rate of recanalization.

## Author Contributions

Conception and design: MV, JH, NH, MK

Analysis and interpretation: MV, NH, MK

Data collection: MV, JH, NH, MK

Writing the article: MV, JH, NH, MK

Critical revision of the article: MV, NH, MK

Final approval of the article: MV, JH, NH, MK

Statistical analysis: MV, MK

Obtained funding: Not applicable

Overall responsibility: MV

## Disclosures

None.
